# Postnatal Growth Velocity and Overweight in Early Adolescents: A Comparison of Rural and Urban African Boys and Girls

**DOI:** 10.1002/ajhb.22575

**Published:** 2014-06-20

**Authors:** ED Chirwa, P Griffiths, K Maleta, P Ashorn, JM Pettifor, SA Norris

**Affiliations:** 1 Wits/MRC Developmental Pathways for Health Research Unit, Department of Pediatrics, Faculty of Health Sciences, University of the WitwatersrandJohannesburg, South Africa; 2 Centre for Global Health and Human Development, Loughborough UniversityLoughborough, Leicestershire, LE 11 3TU, United Kingdom; 3 Department of Community Health, College of Medicine, University of MalawiMalawi; 4 Department of International Health, School of Medicine, University of Tampere, Finland and Department of Pediatrics, Tampere University HospitalFinland

## Abstract

**Objectives:**

To compare growth velocity of two African child cohorts and examine the relationship between postnatal growth velocity in infancy/early childhood and the risk of overweight/stunting in early adolescence.

**Methods:**

The study used data from two child cohorts from urban (Birth to Twenty Cohort, South Africa) and rural (Lungwena Child Survival Study, Malawi) African settings. Mixed effect modelling was used to derive growth and peak growth velocities. *T*-tests were used to compare growth parameters and velocities between the two cohorts. Linear and logistic regression models were used to determine the relationship between growth velocity and early adolescent (ages 9–11 years) body mass index and odds of being overweight.

**Results:**

Children in the BH cohort were significantly taller and heavier than those in the Lungwena cohort, and exhibited faster weight and height growth velocity especially in the first year of life (*P* < 0.05). No significant association was shown between baseline weight (*α*_w_) and overweight in early adolescence (OR = 1.25, CI = 0.67, 2.34). The weight growth velocity parameter *β*_w_ was highly associated with odds of being overweight. Association between overweight in adolescence and weight velocity was stronger in infancy than in early childhood (OR at 3 months = 4.80, CI = 2.49, 9.26; OR at 5 years = 2.39, CI = 1.65, 3.47).

**Conclusion:**

High weight and height growth velocity in infancy, independent of size at birth, is highly associated with overweight in early adolescence. However, the long term effects of rapid growth in infancy may be dependent on a particular population's socio-economic status and level of urbanization. Am. J. Hum. Biol. 26:643–651, 2014. © 2014 The Authors American Journal of Human Biology Published by Wiley Periodicals, Inc.

Several studies have shown the association between early childhood growth and later health outcomes such as diabetes, cardiovascular diseases, and obesity (Cameron and Demerath, 2002; Cameron et al., 2003; Adair, 2007; Adair et al., 2009). In particular, studies have examined the critical periods in infancy and early childhood that are associated with these health outcomes (Black and Krishnakumar, 1999; McCarthy et al., 2007; Botton et al., 2008; Ridgway et al., 2009).

Both growth retardation and rapid growth in the different stages of early life are predictive of the later health outcomes (Cameron and Demerath, 2002; Li, 2007; Cameron et al., 2005; Stein et al., 2010). For example, Flexeder et al. (2012) found that rapid weight gain in infancy is associated with physician-diagnosed asthma in school-aged children (Flexeder et al., 2012). A number of studies have examined the relationship between early growth and later health outcomes in high income as well as low and middle income countries (LMIC; Martorell, 1995; Ong et al., 2000; Li, 2007; Salonen et al., 2009; Mesa et al., 2010; Stein et al., 2010). However, there have also been inconsistent findings regarding the relationship between growth retardation, or stunting and overweight and obesity in later life. Whereas, it has been suggested that childhood under-nutrition predisposes a child to weight gain in later life (Hoffman et al., 2000), other studies have found that childhood stunting was associated with lower body mass index (BMI; Schroeder et al., 1999; Walker et al., 2007).

Several studies have also shown the short term and long term benefits of rapid growth for children in resource-poor settings (Victora et al., 2001; Kalanda et al., 2005; Hoddinott et al., 2008; Victora et al., 2008). The short term benefits include, reduced morbidity and mortality, and improved cognitive development, while long term benefits include improved human capital and improved reproductive outcomes in women.

Although a wide body of evidence supports the long term benefits and detrimental effects of early rapid growth in LMIC, few studies have looked at this relationship in a sub-Saharan African context, due to the limited number of birth cohort studies. Thus, this study compared the growth velocities of two cohorts from rural and urban African settings, and examined the relationship between size at birth (birth weight), growth velocity in infancy and early childhood, and early adolescent obesity. The two cohorts are likely to be at different stages of nutritional transition, considering the rural cohort is from a very low income country while the urban cohort is from a middle income country. Urbanization is generally linked to changes in lifestyle factors that affect obesity risk, such as dietary patterns and sedentary behaviors. Thus, apart from the nutritional differences, there may also be social, cultural, economic and environmental differences between the two cohorts that may affect growth and development of the children and also affect their risk of obesity.

Mixed-effects modelling and childhood structural growth model were used to examine the relationship between postnatal growth velocity and obesity or stunting in early adolescence (ages 9–11 years). Mixed-effects modelling is flexible in dealing with unbalanced longitudinal measurements, and takes into account correlations between repeated measurements. The use of the structural growth curve allowed for the estimation of the growth rates at any given age.

## Subjects and Methods

Weight and height measurements from 2 African longitudinal cohorts were used. The Bone-Health (BH) Study is a sub-sample of the Birth-to-Twenty (BH) birth cohort in Johannesburg, South Africa, which includes 453 black participants. The cohort has anthropometric measurements at birth, 3 months, 6 months, 1 year, 2 years, 4 years, 5 years, 7/8 years, 9 years, and 10 years. Birth weights were extracted from birth records, while subsequent weight/height measurements were obtained using standard anthropometric techniques (Cameron, 1984). More specific details about this urban cohort are reported elsewhere (Cameron et al., 2003; Cameron et al., 2005).

The rural component of the study used the Lungwena Child Survival Study (LCSS), which is a cohort of about 729 children living in Mangochi, a rural district in southern Malawi. The on-going study has growth data of children from birth to 16 or 17 years of age. The anthropometric data in this cohort were collected monthly from birth until 18 months, 3 monthly until 60 months, then at 6 years, 8–9 years, 10 years, 12 years, and 15 years. Weight and height were measured during home visits, using portable spring-scales and self-made length boards, having reading increments of 100 g and 5 mm, respectively. More specific details for the Lungwena cohort are reported elsewhere (Espo et al., 2002; Maleta et al., 2003).

**Figure 1:**
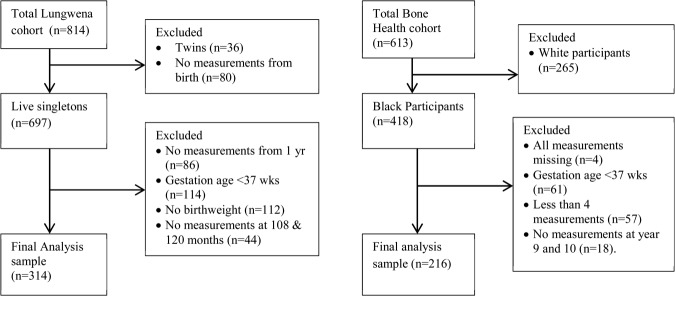
Flowchart of the samples available for analysis from the two cohorts.

In both cohorts, growth velocities were derived from a structural growth model fitted to growth data from birth to 60 months. The exclusion criteria and the overall number of participants available for analysis are shown in Figure 1.

Because of differences in the socio-economic status (SES) variables collected in the two cohorts, an SES score was calculated separately for each cohort. The SES variables in the BH cohort included household assets such as car, TV, fridge and washing machine, and household facilities such as type of water system, toilet type and electricity. The SES variables for the Lungwena cohort included ownership of land, farm animals, bicycle, and radio, amongst others and household variables such as maternal and paternal literacy level. In each cohort, an asset score was initially derived based on household assets. Principal component analysis was then used to derive an overall SES score by combining the asset score with other household and community SES variables.

A Berkey-Reed first order model (Berkey, 1982) was fitted using mixed effects modelling. The model has the functional form;


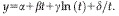


where *y* = weight or height of child at age *t*, the function parameter *α* is related to the baseline weight or height at birth, *β* is related to the linear component of the growth velocity, *γ* is related to the deceleration in growth velocity and *δ* represents an inflection point that allows growth velocity to peak after birth rather than at birth (Mook-Kanamori et al., 2011).

The model was modified as suggested by Simondon et al. (1992), so that it is defined at birth (*t* = 0) as follows:


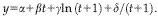


Previous analyses have shown this model to have optimum fit to the BH cohort growth data (Chirwa et al., 2014). The model was used to describe growth patterns in early childhood after adjusting for maternal characteristics (maternal height and age), SES and gestational age. Separate models were fitted for boys and girls in each cohort. The first order derivative of the model was then used to derive weight and height velocities over time (Botton et al., 2008; Mook-Kanamori et al., 2011). The growth velocity function was then used to derive the peak weight velocity (PWV), peak height velocity (PHV) and the age at which a child reached its PWV (APWV) and its peak height velocity (APHV). The primary outcomes were BMI and the proportion of children who were overweight in the 9–11 year age group. BMI cut-off charts for children were used to calculate corresponding overweight cut-offs (Cole et al., 2000,2007).

The derived parameter estimates, growth velocity, infant peak velocity and the age at peak velocity were used as predictors of adolescent BMI or obesity. *T*-tests were used to compare weight, height growth velocity, peak growth velocity between boys and girls within and between cohorts. Linear regression was used to examine the relationship between BMI-for-age *z*-scores (BMIZ) in late childhood and early adolescence (9–11 years) and predictors, adjusting for birth weight, sex and cohort differences. Logistic regression was then used to explore predictors of obesity, adjusting for cohort differences and birth weight. Analysis was done using Stata Version 11, and all statistical tests were performed at 5% significance level.

The BH study was approved by the Human Research Ethics Committee of the University of Witwatersrand, while the LCSS was approved by the Malawi National Health Science Research Committee.

## Results

### Descriptive statistics

The proportion of boys in the BH cohort was higher than that of girls (57% vs. 43%), while the proportion of boys and girls in the Lungwena cohort was almost the same (51% vs. 49%). Of the 216 children in the BH cohort, almost half were first born, while only 19% of the 341 children from the Lungwena cohort were first born. The average maternal age for the BH cohort was 25 years (with standard deviation (SD) of 5.9), while the average age in the Lungwena cohort was 26 years (SD = 6.5). BH cohort mothers were on average taller than their Lungwena counterparts (158 cm vs. 155 cm).

Table1 shows the mean anthropometric measurements in infancy/early childhood and late childhood/early adolescence between boys and girls in the two cohorts. There were no significant differences in the average size at birth between the cohorts (*P* > 0.05). However, BH boys and girls experienced more rapid weight gain from 3 months onwards as shown by the significant differences in mean weight from 3 months, with BH boys and girls weighing on average more than their Lungwena counterparts. Although there were no data on birth length for the BH cohort, subsequent measurements showed BH boys and girls were on average significantly taller than their Lungwena counterparts.

**TABLE 1:** Comparison of physical growth measurements between boys and girls in the two cohorts

	*N*	Boys	Girls		*n*	Boys	Girls	Sig^1^	Sig^2^
Birth and early childhood									
Weight (kg)									
Birth weight	216	3.2 ± 0.5	3.1 ± 0.4		341	3.3 ± 0.5	3.2 ± 0.5	0.115	0.070
3 months	82	6.5 ± 0.8	5.8 ± 0.7		274	6.0 ± 0.8	5.5 ± 0.7	<0.001	<0.001
6 months	52	8.1 ± 0.9	7.4 ± 1.0		286	7.2 ± 1.0	6.6 ± 0.9	0.011	<0.001
1 year	193	9.7 ± 1.4	9.2 ± 1.3		302	8.4 ± 1.1	8.0 ± 1.1	0.012	0.002
2 year	153	11.8 ± 1.7	11.4 ± 1.4		308	10.5 ± 1.4	10.1 ± 1.3	0.126	0.010
4 year	210	15.5 ± 1.9	14.9 ± 1.8		326	14.6 ± 1.5	14.0 ± 1.6	0.021	<0.001
5 year	187	18.6 ± 2.0	17.9 ± 2.1		338	16.0 ± 1.8	15.5 ± 1.9	0.022	0.016
Height (cm)									
Birth length	–	–	–		341	48.9 ± 2.2	47.9 ± 2.1	-	<0.001
3 months	82	60.8 ± 2.9	58.7 ± 2.8		274	57.3 ± 2.4	56.1 ± 2.6	0.001	<0.001
6 months	52	66.3 ± 2.8	63.4 ± 4.1		286	62.5 ± 2.6	60.6 ± 2.6	0.015	<0.001
1 year	187	74.4 ± 3.1	72.6 ± 3.0		302	69.0 ± 2.6	67.8 ± 2.6	<0.001	<0.001
2 years	143	83.6 ± 3.7	82.2 ± 3.1		308	78.2 ± 3.7	76.9 ± 3.4	0.010	0.002
4 years	210	99.2 ± 3.9	97.9 ± 3.9		326	93.9 ± 4.3	92.4 ± 4.4	0.017	0.002
5 years	187	108.8 ± 4.0	107.4 ± 4.2		338	100.9 ± 4.4	99.4 ± 4.7	0.022	0.003
Early adolescence									
Age (years)	216	10.5 ± 0.3	10.5 ± 0.3		341	10.3 ± 0.3	10.35± 0.3	<0.001	0.011
Height (cm)	216	137.8 ± 6.1	138.4 ± 5.9		341	129.7± 5.5	128.46 ± 6.0	<0.001	<0.001
Weight (kg)	216	32.9± 6.2	33.4 ± 6.6		341	25.7 ± 3.2	25.22 ± 3.5	<0.001	<0.001
BMI (kg/m^2^)	216	17.3± 2.5	17.4 ± 2.9		341	15.3 ± 1.2	15.22 ± 1.2	<0.001	<0.001
Overweight (%)		26(21%)	22(24%)			2(1.2%)	0(0%)	<0.001	<0.001
Underweight (%)		1(0%)	5(5%)			13(8)	17(10%)	<0.001	0.246

Sig^1^: BH boys vs. Lungwena boys. All mean comparisons done using *t*-test.

Sig^2^: BH girls vs. Lungwena girls. All proportions comparisons done using Fishers' exact test.

**Figure 2:**
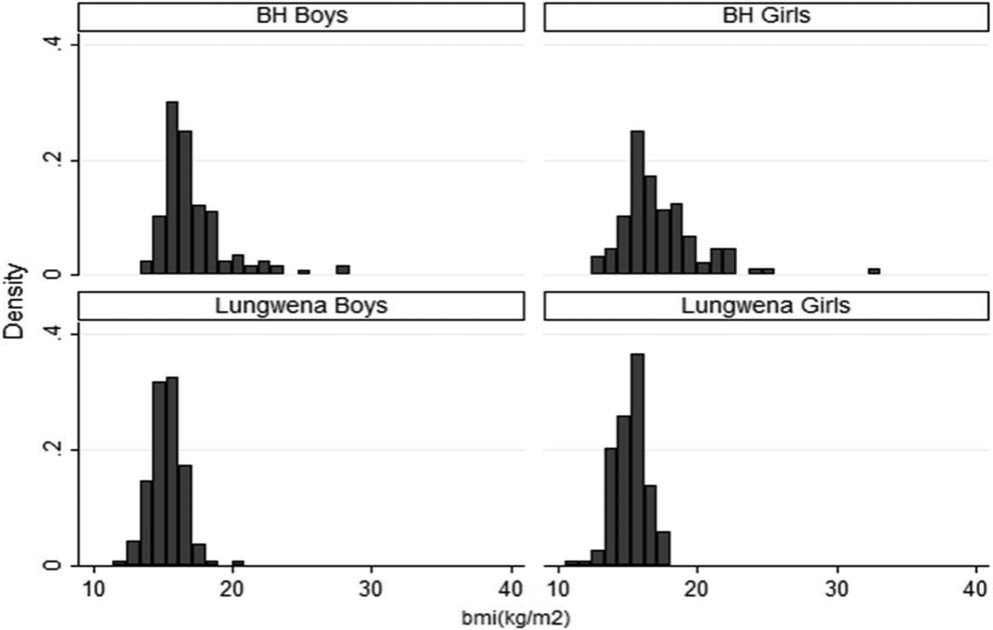
BMI in early adolescence for boys and girls in the two cohorts.

In early adolescence, there were no significant differences in weight, age, height or BMI between boys and girls within each cohort, but there were significant differences between the cohorts, with BH boys and girls having higher mean BMI, height and weight compared to the Lungwena boys and girls. However, unlike the pattern in infancy/early childhood, girls in the BH cohort were on average taller and weighed more than boys at ages 9/10 years. Figure 1 shows the distribution of BMI for boys and girls in the two cohorts, with BH children having largely higher BMI than Lungwena children. However, there is also wider variation in the BMI values in the BH cohort (Fig. 2).

### Average model parameter and growth velocity estimates

Parameter estimates for weight and height models were derived for each child using the random components of the mixed models. A *t*-test comparison of these parameter estimates showed significant differences in the average parameter estimates between BH boys and Lungwena boys for both the height and weight growth model (Table2). Similar results were also found amongst the girls from the two cohorts. BH boys had the highest starting values (*α*_w_ and *α*_h_), as well as the highest linear growth rates (*β*_w_ and *β*_h_). However the *α*_h_ for the height model for both cohorts represents starting height/length at 3 months, due to the BH cohort not having height/length measurements at birth. Within each cohort, there generally were no significant differences in model parameter estimates between boys and girls in both cohort except for *β*_w_ and *β*_h_ in the Lungwena cohort. Non-significant difference in the linear component of the velocity curve between boys and girls in the Lungwena cohort was also shown by the non-significant differences in both weight and height velocities. There were significant differences in the weight velocity between boys and girls in the BH cohort except at 24 months. Although boys in the Lungwena cohort tended to have higher weight velocities than girls, there were no significant differences in average weight velocity between boys and girls in the Lungwena cohort from 12 months onwards. Boys in the BH cohort generally exhibited higher height velocities than girls, with significant differences in the average height velocity between boys and girls in the first 2 years of life (*P* < 0.001).

**TABLE 2:** Average differences in parameter estimates, growth velocity and peak velocity between cohort and sex

	BH cohort		Lungwena cohort		Coh. dif.		Sex dif.	
	Boys	Girls	Boys	Girls	M	F	BH	LUN
	Mean (SD)	Mean (SD)	Mean (SD)	Mean (SD)	sig	sig	sig	Sig
Parameters (weight)								
*α*_w_	6.70 (0.45)	4.44 (0.34)	5.49 (0.66)	5.13 (0.63)	a	a	a	a
*β*_w_	0.16 (0.02)	0.13 (0.03)	0.14 (0.02)	0.14 (0.03)	a	0.023	a	0.816
*γ*_w_	0.42 (0.02)	1.16 (0.03)	0.54 (0.02)	0.48 (0.03)	a	a	a	a
*δ*_w_	−3.50 (0.03)	−1.36 (0.03)	−2.68 (0.03)	−2.43 (0.03)	a	a	a	a
Parameters (height)								
*α*_h_	66.5 (2.07)	56.4 (1.95)	54.1 (2.28)	53.5(2.20)	a	a	a	0.030
*β*_h_	0.59 (0.04)	0.52 (0.06)	0.52 (0.06)	0.52 (0.07)	a	0.815	a	0.654
*γ*_h_	1.37 (0.03)	4.42 (0.06)	3.79 (0.07)	3.56 (0.08)	a	a	a	a
*δ*_h_	−39.8 (0.04)	−22.6 (0.05)	−15.3 (0.06)	−16.8 (0.08)	a	a	a	a
Peak velocity								
PWV	1.31 (0.26)	1.39 (0.28)	1.36 (0.24)	1.35 (0.29)	0.067	0.217	0.024	0.579
APWV (months)	2.49 (0.02)	2.86 (0.05)	3.10 (0.06)	2.96 (0.07)	a	a	a	a
PHV	5.77 (0.37)	5.59(0.58)	5.51 (0.67)	5.53 (0.84)	a	0.556	0.006	0.882
APHV (months)	5.00 (0.08)	7.24 (0.34)	6.92 (0.47)	5.43 (0.27)	a	a	a	a
Weight velocity (kg/months)								
3 months	0.48 (0.03)	0.50 (0.03)	0.44 (0.03)	0.41 (0.03)	a	a	a	a
6 months	0.29 (0.03)	0.33 (0.03)	0.27 (0.03)	0.26 (0.03)	a	a	a	a
12 months	0.21 (0.03)	0.23 (0.03)	0.20 (0.03)	0.19 0.03)	a	a	a	0.056
24 months	0.18 (0.02)	0.18 (0.03)	0.17 (0.02)	0.17 (0.03)	a	a	0.795	0.431
48 months	0.17 (0.02)	0.16 (0.03)	0.15 (0.02)	0.15 (0.03)	a	0.103	0.002	0.796
60 months	0.17 (0.02)	0.15 (0.03)	0.15 (0.02)	0.15 (0.03)	a	0.540	a	0.873
Height velocity (cm/monthso)								
3 months	3.34 (0.17)	2.99 (0.14)	2.41 (0.10)	2.45 (0.11)	a	a	a	0.002
6 months	1.61 (0.07)	1.62 (0.07)	1.37 (0.08)	1.37 (0.09)	a	a	0.221	0.468
12 months	0.94 (0.05)	1.00 (0.07)	0.91 (0.07)	0.89 (0.09)	a	a	a	0.144
24 months	0.71 (0.04)	0.73 (0.06)	0.70 (0.07)	0.69 (0.09)	0.134	a	a	0.211
48 months	0.64 (0.04)	0.63 (0.06)	0.61 (0.06)	0.60 (0.08)	a	0.015	0.019	0.352
60 months	0.63 (0.04)	0.60 (0.06)	0.58 (0.06)	0.58 (0.08)	a	0.123	a	0.393

M = BH boys vs. Lungwena boys

F = BH girls vs. Lungwena girls

BH = BH boys vs. BH girls

LUN = Lungwena boys vs. Lungwena girls

PWV= Peak weight velocity (kg/months)

APWV = Age at peak weight velocity (month)

PHV= Peak height velocity (cm/months)

APHV= Age at peak height velocity (month)

All comparisons done using the *t*-test.

a : *P*-value <0.001.

As expected and consistent with the changes in average weight and height over time, as shown in Table1, weight and height velocities were highest in the first 12 months, with growth rates rapidly declining from 12 months (Table2). There were no significant differences in the average weight velocities between small for gestational age infants (WAZ at birth < −2) and appropriate for gestational age (AGA) infants (data not shown). Similar results were found when comparing those with low birth weight (birth weight <2.5 kg) to those with normal birth weight (data not shown).

The significant differences in the parameter estimates between BH boys and Lungwena boys as well as between girls in the two cohorts, were also shown by the significant differences in the weight and height velocities between the cohorts, with the BH boys having higher growth rates than their Lungwena counterparts. Similarly, BH girls exhibited higher growth rates than Lungwena girls. A strong positive linear relationship between weight and height velocity (*r* = 0.89, *P* < 0.001) was observed.

BH girls had the highest infancy PWV (1.39 kg/month). However, no significant difference was observed in the infancy PWV or PHV between sexes within the Lungwena cohort or as well as between girls in the two cohorts. BH boys which had the smallest PWV also had the youngest age at APWV. BH boy's height velocity also peaked earliest compared to the other three groups.

### Relationship between birth weight, growth velocity, peak velocity and adolescent BMI

There were no significant correlations between birth weight and weight velocity (*r* = −0.08, *P* = 0.06) or height velocity (*r* = −0.05, *P* = 0.27).

There was a positive but weak relationship between birth weight and adolescent BMI, with birth weight only explaining 1% of the variation in BMI (Table3). This relationship did not change even after adjusting for sex differences. However, when cohort differences were taken into account, the total variation explained by the model increased from 1% to 24%, signifying the large differences that exist between the two cohorts. The effect of birth weight on BMI was also supported by the results from the relationship between *α*_w_ for the weight model and BMI, with no significant sex difference being observed. Within each of the three models using parameter estimates from weight models, *β*_w_ had the strongest linear relationship with BMI compared to the other three parameter estimates, with no linear relationship being observed between adolescent BMIZ and *δ*_w_. Both *γ*_w_ and *δ*_w_ were non-significant when effect of cohort and sex differences as well as birth weight, were taken into account.

**TABLE 3:** Relationship between adolescent BMI and growth velocity, peak velocity and Reed1 model parameters, adjusting for sex and cohort differences

Main predictor	Model 1		Model 2		Model 3	
	*β* (SE)	*R*^2^	*β* (SE)	*R*^2^	*β* (SE)	*R*^2^
Sex: boys	Ref					
girls	−0.18 (0.09)^a^	0.01				
Cohort: BH	Ref					
LUN	−1.07 (0.08)	0.22				
Birth weight	0.27 (0.10)	0.01				
Parameters (weight)						
*α*_w_	0.34 (0.05)	0.08	0.33 (0.05)	0.08	0.25 (0.06)^a^	0.27
*β*_w_	21.2 (1.55)	0.25	21.3 (1.61)	0.25	18.9 (1.45)	0.42
*γ*_w_	0.96 (0.18)	0.05	1.00 (0.18)	0.07	0.31 (0.21)^a^	0.25
*δ*_w_	−0.05 (0.07)^a^	0.001	−0.04 (0.07)^a^	0.01	0.06 (0.09)^a^	0.25
Parameters (height)						
*α*_h_	0.08 (0.01)	0.15	0.07 (0.01)	0.15	0.001(0.013)^a^	0.25
*β*_h_	4.32 (0.62)	0.08	4.18 (0.63)	0.08	2.20 (0.60)	0.26
*γ*_h_	−0.24 (0.04)	0.05	−0.24 (0.04)	0.06	0.017 (0.05)^a^	0.25
*δ*_h_	−0.05 (0.01)	0.16	−0.05 (0.002)	0.18	0.001 (0.01)^a^	0.25
Infant peak velocity						
PWV	1.85 (0.16)	0.20	1.84 (0.16)	0.20	1.86 (0.14)	0.44
PHV	0.35 (0.07)	0.05	0.33 (0.07)	0.05	0.23 (0.06)	0.26
APWV	−1.33 (0.19)	0.08	−1.39 (0.19)	0.10	1.32 (0.29)	0.27
APHV	−0.004 (0.05)^a^	0.001	−0.008 (0.05)^a^	0.01	0.05 (0.04)^a^	0.24
Weight velocity						
3 months	14.8 (0.80)	0.38	14.7 (0.81)	0.38	12.3 (1.05)	0.40
6 months	18.3 (1.03)	0.36	18.2 (1.04)	0.36	14.8 (1.23)	0.40
12 months	21.7 (1.28)	0.34	21.7 (1.31)	0.34	17.4 (1.37)	0.42
24 months	23.5 (1.43)	0.33	23.7 (1.47)	0.33	19.3 (1.42)	0.43
48 months	23.3 (1.50)	0.30	23.5 (1.55)	0.30	19.6 (1.44)	0.43
60 months	22.5 (1.52)	0.29	23.1 (1.57)	0.29	19.5 (1.44)	0.43
Height velocity						
3 months	1.31(0.10)	0.24	1.32 (0.10)	0.25	0.81 (0.25)	0.26
6 months	3.68 (0.29)	0.22	3.68 (0.29)	0.24	1.86 (0.50)	0.26
12 months	3.79 (0.54)	0.08	3.72 (0.55)	0.09	1.50 (0.54)	0.26
24 months	3.67 (0.65)	0.05	3.54 (0.65)	0.06	2.22 (0.59)	0.26
48 months	4.07 (0.66)	0.06	3.93 (0.66)	0.07	2.32 (0.61)	0.26
60 months	3.98 (0.65)	0.06	3.83 (0.67)	0.07	2.30 (0.61)	0.26

a Effect of main predictor was not significant.

Model 1: Adolescent BMI vs. main predictor.

Model 2: Adolescent BMI vs. main predictor (adjusting for birth weight). Birth weight was non-significant when adjusted for the main predictors in Model 2.

Model 3: Adolescent BMI vs. main predictor (adjusting for birth weight, sex and cohort differences).

*R*^2^ = total variation in BMI explained by the overall model.

There was a negative linear relationship between adolescent BMIZ and APWV, indicating that infants that reached their PWV early were more likely to have high BMI in adolescence. A strong negative correlation was also observed between PWV and APWV (*r* = −0.53, *P* < 0.001), indicating that infants with low PWV were more likely to reach their peak later than infants that exhibited high PWV. There was no significant linear relationship between adolescent BMIZ and age at which the infant reached PHV.

There was a general decrease in the relationship between weight velocity and adolescent BMIZ over time even after adjusting for birth weight (*R*^2^_(3m)_ = 0.38, *R*^2^_(60m)_ = 0.29).

Even though there was a strong relationship between adolescent BMIZ and height velocity in the first 6 months, even after adjusting for birth weight, as observed from the *R*^2^ values (models 1 and 2), there were no differences in the strength of the relationship over time when cohort and sex differences were taken into account (model 3).

### Relationship between birth weight, growth velocity, peak velocity and adolescent obesity

Table4 shows the association between adolescent overweight and growth velocity. The study found no association between sex and being overweight adolescent, even though girls had lower odds of being overweight compared to boys (OR = 0.88, *P* = 0.671). The Lungwena cohort had lower odds of being overweight than the BH cohort (OR = 0.02, *P* < 0.001). The odds did not change even after adjusting for sex differences. Despite the odds of being overweight increasing with increase in birth weight, the association was not significant (OR = 1.25, *P* = 0.486).

**TABLE 4:** Odds ratios from the relationship between overweight and growth velocity, peak velocity and Reed 1 parameters, adjusting for sex and cohort difference

Main predictor	Model 1	Model 2	Model 3^a^
	OR (95% CI)	OR (95% CI)	OR (95% CI)
Sex: boys	Ref		
:girls	0.88 (0.49, 1.58)^b^		
Cohort: BH	Ref		
LUN	0.02 (0.005, 0.09)		
Birth weight	1.25 (0.67, 2.34)^b^		
Parameters (weight)			
*α*_w_	1.53 (1.19, 1.97)	1.55 (1.20, 2.02)	1.12 (0.89, 1.40)^b^
*β*_w_	2.21 (1.65, 2.96)	2.27 (1.68, 3.08)	2.05 (1.46, 2.87)
*γ*_w_	1.46 (1.24, 1.72)	1.48 (1.25, 1.75)	1.08 (0.92, 1.27)^b^
*δ*_w_	1.06 (0.87, 1.29)^b^	1.07 (0.88, 1.31)^b^	1.05 (0.92, 1.21)^b^
Parameters (height)			
*α*_h_	1.79 (1.46,2.21)	1.79 (1.45, 2.22)	0.88 (0.67, 1.15)^b^
*β*_h_	1.71 (1.21, 2.42)	1.70 (1.20,2.41)	1.14 (0.77, 1.70)^b^
*γ*_h_	0.74 (0.62, 0.89)	0.74 (0.62, 0.89)	1.05 (0.90, 1.24)^b^
*δ*_h_	0.53 (0.43, 0.65)	0.53 (0.43, 0.65)	1.10 (0.84, 1.45)^b^
Infancy peak velocity			
PWV	1.42 (1.08, 1.87)	1.42 (1.07, 1.88)	1.68 (1.17, 2.42)
PHV	1.58 (0.99, 2.52)^b^	1.56 (0.97, 2.50)^b^	1.44 (0.76, 2.74)^b^
APWV	0.06 (0.01, 0.12)	0.03 (0.01, 0.12)	4.31 (0.78, 23.7)^b^
APHV	0.99 (0.74, 1.35)^b^	0.99 (0.74, 1.34)^b^	1.17 (0.88, 1.55)^b^
Weight velocity			
3 months	7.49 (4.50, 12.46)	7.96 (4.68, 13.52)	4.80 (2.49, 9.26)
6 months	4.07 (2.82, 5.86)	4.09 (2.84, 5.90)	2.60 (1.77, 3.83)
12 months	3.51 (2.48, 4.94)	3.58 (2.52, 5.08)	2.46 (1.89, 3.61)
24 months	3.06 (2.20, 4.25)	3.18 (2.26, 4.47)	2.44 (1.68, 3.55)
48 months	2.67 (1.95, 3.66)	2.78 (2.01, 3.86)	2.41 (1.68, 3.64)
60 months	2.60 (1.90, 3.56)	2.71 (1.96, 3.75)	2.39 (1.65, 3.47)
Height velocity			
3 months	3.02 (2.19, 4.15)	3.01 (2.19, 4.14)	0.87 (0.50, 1.50)^b^
6 months	5.49 (3.27, 9.22)	5.52 (3.29, 9.24)	1.62 (0.61, 4.32)^b^
12 months	2.25 (1.60, 3.15)	2.25 (1.60, 3.16)	1.35 (0.89, 2.03)^b^
24 months	1.55 (1.13, 2.14)	1.54 (1.12, 2.13)	1.34 (0.88, 2.04)^b^
48 months	1.51 (1.10, 2.09)	1.50 (1.09, 2.07)	1.32 (0.86, 2.03)^b^
60 months	1.55 (1.12, 2.14)	1.53 (1.11, 2.13)	1.26 (0.82, 1.94)^b^

a Not adjusted for sex due to limited number of children in Lungwena cohort with outcome.

b Effect of main predictor was not significant.

Model 1: Overweight vs. main predictor.

Model 2: Overweight vs. main predictor (adjusting for birth weight).

Model 3: Overweight vs. main predictor (adjusting for birth weight and cohort differences).

Consistent with the observed relationship between BMI and linear growth rates in weight (*β*_w_) as shown in Table3, the study also found strongest association between being overweight and linear growth rate in weight (*β*_w_), even after adjusting for cohort differences and birth weight (OR = 2.05, *P*-value < 0.001). While there was a strong association between being an overweight adolescent and a child's estimated baseline weight (*α*_w_), this relationship was not significant when cohort differences were taken into account. After adjusting for cohort differences and birth weight, only the linear growth rate function (*β*_w_) was found to be associated with adolescent overweight.

The study also found strong association between adolescent overweight and linear growth rates in height, with children exhibiting faster height growth rates being more likely to be overweight in adolescence. However, this relationship was non-significant when cohort differences were taken into account. Consistent with weight model parameters, baseline height (*α*_h_) and the decrease in height velocity over time (*γ*_h_) were also not associated with being overweight.

The logistic regression models showed a stronger association between overweight in early adolescence (ages 9–11 years) and weight gain in infancy than with weight gain in early childhood. At 3 months, every 1 SD increase in weight velocity had an eightfold odds of being overweight in early adolescence. These odds reduced with age such that by the time a child is 5-years-old, every 1 SD increase in weight velocity resulted in almost threefold odds of being overweight. Despite there being a decrease in the odds of being overweight after adjusting for cohort differences and birth weight, the trend over time was the same. The same trend was observed with height velocity. However, there were no significant association between height velocity and being overweight after adjusting for birth weight and cohort differences. No association was found between obesity and PHV, age at PHV or age at PWV, when adjusted for cohort difference. However children with high PWV were more likely to be overweight in adolescence even after adjusting for cohort differences.

## Discussion

This study has been able to demonstrate a positive linear relationship between rapid weight gain in infancy and early childhood and early adolescent BMI, as well as shown a relationship between rapid growth in the early years and the odds of being overweight/obese in early adolescence.

The high odds ratio and *R*^2^ values between growth velocity in the first year of life (the period which was also characterised by high growth velocity) and adolescent BMI, highlight the association between rapid weight gain and obesity in early adolescence. The decreasing trend in the OR values in later early childhood highlights the critical period during infancy that is highly associated with adolescence/adult obesity. This supports what prior studies in the BH cohort and others elsewhere have found, albeit using different methods or measures (Cameron et al., 2003; Ekelund et al., 2007; McCarthy et al., 2007; Botton et al., 2008; Adair et al., 2009; Demerath et al., 2009; Stein et al., 2010). In a study of the relationship between rapid weight gain in the first 2 years of life and obesity in childhood in BH children with appropriate birth weight for gestational age (AGA), Cameron et al. (2003), using weight-for-age *z*-scores, found that children that exhibited rapid growth in infancy were significantly taller, and weighed more in childhood. Our study has been able to demonstrate this using parameter estimates from the Reed1 model, with the parameter *β*_w_, a function related to growth velocity being highly positively associated with early adolescent BMI. Consistent with the study by Mook-Kanamori et al., our study found high PWV to be highly associated with early adolescence overweight. The significance of the relationship between rapid weight gain in infancy and adolescent BMI was also highlighted by the negative associated between APWV and adolescent BMI, indicating that infants that reached infant PWV early were more likely to have high BMI. Apart from that, our study has also explored the relationship using height velocity and extended the period to early adolescence (9–11 years). Our study has also been able to show similar association between rapid growth and adolescent BMI in a rural population, which is from a predominately malnourished population, with high levels of stunting and underweight. The critical period of development is the same in both cohorts. However, the rapid infant growth in this rural population seems to have beneficial effects, as it protects the adolescent child from the effects of under-nutrition**,** with few cases of obesity.

There are several hypothesised biological relationships between prenatal and postnatal growth and obesity in later life, and a large body of evidence supports these hypothesised relationships (Ong and Loos, 2006; Adair, 2007; Ekelund et al., 2007; Jones-Smith et al., 2007; McCarthy et al., 2007; Chomtho et al., 2008; Druet et al., 2012). These previous studies showed that either small size at birth, small size at birth combined with fast growth or fast growth itself, have effects on later life health outcomes. Studies have also shown that low-birth-weight infants usually exhibit rapid growth during the first year of life (Ong, 2006; Adair, 2007; Johnson et al., 2012). However, our study which used data from two cohorts from different settings in terms of environmental and socio-economic factors, found no relationship between size at birth (birth weight) and growth velocity in both cohorts. Similarly, we found no association between size at birth and overweight in early adolescence. Both birth weight and its estimated parameter (*α*_w_) were not associated with adolescent overweight. The non-significant relationship between birth weight and growth velocity as well as with overweight in early adolescence could also be due to the limited range of birth weight measurements, since our sample excluded preterm babies. Even though there was a wide variation in the age of initial weight measurements for the Lungwena cohort, for babies not delivered in a health facility, the mixed effects model adjusted for the age at which the measurements were taken.

However, the effect of the differences in the environmental and socio-economic factors in the two cohorts were shown by the differences in the growth rates and the postnatal prevalence of stunting/underweight and overweight in the cohorts as well as the significance of cohort term in the models. Despite there being no significant differences in birth weights between the two cohorts, the urban BH children exhibited more rapid weight gain in the first year of life. This rapid weight gain was associated with a high prevalence of overweight adolescents in this population. The differences in the prevalence of overweight adolescents in the two cohorts, considering the non-significant differences in their size at birth, highlights the significance of rapid weight gain rather than birth size, in the relationship between early growth and obesity in adolescence, in this particular setting. These results are in support of the “fast growth and obesity” hypothesis, rather than the “size at birth” or the “size at birth and fast growth” hypotheses. The relationship between faster growth velocity and obesity/overweight in later life, independent of birth weight, has been hypothesised to be mainly due to over-nutrition (Jones-Smith et al., 2013). The more rapid weight gain in the BH cohort relative to the Lungwena cohort may be due to nutritional and environmental differences, among other factors. The slower weight velocity in the Lungwena cohort, from as early as 3 months, could be due to poor maternal nutritional status and the early introduction of complementary foods. As Lungwena is predominately a poor rural community, the complementary foods used are likely to be of poor nutritional content and to expose the infants to pathogens (Espo et al., 2002).

Our results are in general consistent with study by Adair et al. (2013), which also looked at association between weight and height gain, and adult health outcomes in five cohorts from LMIC (Adair et al., 2013). The study found a positive relationship between weight gain and adult BMI, with the strength of the relationship increasing with age at which measurement was taken. However, unlike our study, they also found positive relationship between birth weight and adult BMI, and they also found a decreasing relationship between height gain and BMI. The variations in these results could be due to the differences in the age ranges used as well as the limited amount of observations at 3 and 6 months in the BH cohort of our study.

Apart from looking at cohorts from different SES and environmental settings, the other strength of this study is in the use of mixed effects modelling to model growth trajectories and to derive growth velocities. Mixed effects modelling allowed us to compare growth velocity at any age even though some of the data collection waves in the two cohorts were at different times. Our results are in general, consistent with results from other studies that have used mixed effects. In a study of Dutch children, Mook-Kanamori et al., also using the Berkey-Reed model and mixed effects modelling, found that rapid weight gain in the first months was more associated with risk of overweight than catch-up growth (‘size at birth and fast growth hypothesis’) during the first 2 years (Mook-Kanamori et al., 2011). Similarly, Botton et al. (2008) using the adapted Jenns-Bayley model and using mixed effects modelling, also found increased risk of obesity due to rapid growth in the first 6 months in French children. However, their study also found that this risk started increasing again from 3 years. However, they derived their growth velocity from a model fitted from birth to 10 years, which may have made it possible to pick out the increase in growth velocity from 3 years. Our study fitted the growth model up to 5 years only.

The main limitation of the study is unavailability of data on adolescent factors associated with BMI in one of the cohort, which could have been adjusted for in the relationship between postnatal growth and adolescent BMI/overweight. The other limitation for the study is the amount of missing weight and height measurements during the first year of life in the BH cohort which could have affected the fitness of the growth model used for estimating growth velocity.

In conclusion, although our results support the hypothesis that rapid growth in infancy increases the risk of overweight/ obesity in later life, the long term effects of infancy rapid growth are dependent on the particular population's stage of nutrition transition. For a population in early stages of nutrition transition or with poor nutritional status, rapid growth in early childhood may have long term beneficial effects as was evidenced by the almost non-existent prevalence of overweight in the Lungwena cohort, despite some children exhibiting rapid growth in early childhood. Conversely, for populations undergoing rapid nutrition transition as is the case with the BH cohort, rapid growth has detrimental long term effects, as was evidenced by the prevalence of overweight and obesity in early adolescence. To further explore the relationship between postnatal growth velocity and later health outcome, we would recommend modelling growth into adolescence and also include pubertal stages and SES factors during adolescence that are highly associated with BMI, such as dietary patterns and physical activity behaviors of the adolescents in the cohorts. Further studies in similar cohorts in LMIC might also help in explaining the effect of shifts in dietary and sedentary behaviors associated with urbanization.
